# Efficacy of Magnesium Sulphate as an Adjunct to Lignocaine in Inferior Alveolar Nerve Block for Extraction of Mandibular Third Molar—A Split-Mouth Double-Blinded Randomized Controlled Trial

**DOI:** 10.1155/tswj/3695623

**Published:** 2025-05-19

**Authors:** Anupam Singh, Murali Venkata Rama Mohan Kodali, Kalyana Pentapati, Mehul Saha, Srikanth Gadicherla, Komal Smriti

**Affiliations:** ^1^Department of Oral and Maxillofacial Surgery, Manipal College of Dental Sciences, Manipal, Manipal Academy of Higher Education (MAHE), Manipal, Karnataka, India; ^2^Department of Oral and Maxillofacial Surgery, College of Dentistry, King Faisal University, Al-Ahsa, Saudi Arabia; ^3^Department of Public Health Dentistry, Manipal College of Dental Sciences, Manipal, Manipal Academy of Higher Education (MAHE), Manipal, Karnataka, India; ^4^Department of Oral Medicine and Radiology, Manipal College of Dental Sciences, Manipal, Manipal Academy of Higher Education (MAHE), Manipal, Karnataka, India

**Keywords:** analgesia, disimpaction, lignocaine, magnesium sulphate, third molar surgery

## Abstract

**Background:** Extensive ongoing research is aimed at enhancing the efficacy of inferior alveolar nerve block (IANB). Even though magnesium itself is not a primary analgesic, it has been shown to increase the effects of analgesics when used as an adjuvant or a supplement. Magnesium sulphate (MgSO_4_) has reportedly been used to supplement regional blocks and spinal anaesthesia in various surgical procedures. Building on the concept of MgSO_4_ as an analgesic adjuvant, our study aimed to assess its efficacy in increasing IANB success and controlling postsurgical pain. The split-mouth study evaluated the effectiveness of adding MgSO_4_ to 2% lignocaine in improving block success and offering postsurgical pain relief after the transalveolar extraction of impacted mandibular third molars (MTMs).

**Methodology:** We carried out a double-blinded, randomized, split-mouth study in 26 patients having bilateral impacted MTM. Patients presenting with impacted MTM bilaterally with a Pederson's score of ≤ 6 were included. The primary outcomes evaluated were the onset time, duration of anaesthesia, need for additional injections, and burning sensation during injection. The secondary outcomes assessed included postoperative pain relief and the quantity of rescue analgesics required.

**Results:** The test side showed a significantly longer duration of analgesia than the control side (*p* < 0.001). The MgSO_4_ group showed a lesser requirement for additional injections; however, it was not found to be statistically significant (0.289). No significant differences were seen in postoperative pain and the number of rescue analgesics.

**Conclusion:** The addition of MgSO_4_ to 2% lidocaine resulted in a significantly longer duration of analgesia.

**Trial Registration:** CTRI identifier: CTRI/2018/05/013842

## 1. Background

Mandibular third molar (MTM) disimpaction surgery is a routinely performed minor oral surgical procedure. The success of surgical removal of MTM relies heavily on the use of local anaesthesia (LA) by using inferior alveolar nerve block (IANB) [1]. Nevertheless, the success rate of this nerve block is variable, and in many cases, it fails even when administered by a skilled professional [2].

The effectiveness of LA is greatly influenced by the characteristics of the anaesthetic agent and the techniques used for injection [3]. Typically, effectiveness has been evaluated using indirect measures like the requirement for supplemental injections (reanaesthesia), the total amount or quantity of anaesthesia administered, or the level of intraoperative pain, among other factors [4–6]. Further, the postoperative pain and discomfort results in overall unpleasant experience for the patient and thereby causes apprehension among the patients [7]. The LA's effect lasts about 40–65 min, that is, approximately the time duration of most of the transalveolar surgical procedure requiring MTM disimpaction. Once the local anaesthetic's effect wears off, the patient starts to feel pain and often necessitates the repeat of IANB [8].

Inflammation or pre-existing infection at the surgical site can also influence the success or efficacy of IANB. Inflammation leads to inadequate anaesthesia because inflammatory mediators can activate pain receptors even at minimal levels of stimulation [9]. Inflammation results from the synthesis of prostaglandins, which are generated by the action of cyclooxygenase enzymes on arachidonic acid in cell membranes. These prostaglandins play a role in initiating and intensifying pain. Inflammation causes increased sensitization of nociceptors, which reduces the efficacy of IANB [10, 11].

Extensive ongoing research is focused on improving the effectiveness of IANB [12]. Magnesium's anti-inflammatory effects and its analgesic potency has been studied extensively in postsurgical pain management [13]. Systemic administration of magnesium sulphate (MgSO_4_) has shown to lower the postsurgical analgesic consumption [14, 15]. MgSO_4_ is utilized in anaesthetic practice to enhance the quality of anaesthesia and pain relief [16]. Adding MgSO_4_ to lidocaine in regional anaesthesia showed a prolonged duration of analgesia and overall reduction in failure rate [17]. When used intrathecally, magnesium acts as an adjuvant to local anaesthetic and boosts the analgesic effect of spinal anaesthesia and epidural anaesthesia for postsurgical pain management [18]. In gynecology patients undergoing total intravenous anaesthesia, intravenous MgSO_4_ reduced the need for rocuronium and improved the quality of postsurgical analgesia [19]. Similarly, studies have shown that local administration of MgSO_4_ to the surgical area can also decrease postoperative analgesia requirements [20].

Previous trials on the use of MgSO_4_ with lignocaine for the effectiveness of nerve blocks in patients with irreversible pulpitis have shown a marked improvement in the quality and success of anaesthesia [21, 22]. These studies were done on patients requiring endodontic treatment with symptoms of irreversible pulpitis. These studies were parallel-arm trials involving separate individuals, and since pain is a subjective measure, it can vary significantly among participants. Hence, through this randomized trial, we aimed to evaluate the effectiveness of IANB administered using lignocaine (1:200,000 adrenaline) with and without the addition of MgSO_4_ for the removal of MTM. The objective was to compare the duration of anaesthesia, the requirement of additional anaesthetic injections, postoperative pain, and the quantity of rescue analgesics required between the IANB administered using lignocaine (1:200,000 adrenaline) with and without the addition of MgSO_4_. The null hypothesis stated that there would be no significant difference in the duration of analgesia, postoperative pain, and the number of rescue analgesics between IANB administered using lignocaine (1:200,000 adrenaline) with and without the addition of MgSO_4_.

## 2. Methodology

The study was conducted adhering to the Declaration of Helsinki for medical protocol and ethics and received approval from the ethics committee of our Hospital (“KH-IEC”: 39/2018). All patients provided prior informed consent before enrolling in the study. A double-blinded, split-mouth randomized controlled trial (RCT) was carried out with 26 patients needing the extraction of bilateral impacted MTMs. The trial was carried out in the unit of oral and maxillofacial surgery from January 2019 to December 2022. The CONSORT guidelines were followed for the design and implementation of the study [23].

Adult patients aged less than 45 years, requiring bilateral impacted MTM extractions via the surgical or transalveolar method, and having a Pederson's difficulty index of less than or equal to 6, were included. Participants with a history of substance abuse, local anaesthetic drug allergy, or patients with uncontrolled medical condition/s (ASA II or more) were excluded.

While assessing pain response, various potential confounders were considered, such as individual baseline pain threshold, psychological factors, and gender differences. To overcome these confounding factors, a split-mouth study design was employed, where each patient acted as their own control. A coin toss was done for randomization to decide the side that received the test or control intervention. All the surgical extractions were performed by a single operator (A.S.). The randomization and allocation of patients were done by one of the two surgeons (S.G. or M.S.). Both the operator administering the local anaesthetic and the patient were blinded to the intervention.

Sample size was estimated using Gpower software (Version 3.1.9.4 Universität Kiel, Germany) based on the intraoperative pain (visual analogue scale (VAS)) reported by Mousavi et al., which yielded an effect size of 1.38 [22]. Using this effect size, with a power of 90% and an alpha error of 5%, the sample size was estimated to be 24. Considering an attrition of 10%, the sample size was inflated and rounded to 26.

### 2.1. Solution Preparation

Two different solutions of LA were used in this trial. “Solution A” was made of a modified solution of lignocaine 2% with adrenaline 1:200,000 (Neon Pharma Ltd., India) mixed with 50% *w*/*v* MgSO_4_ (Medilife Health Sciences Pvt. Ltd., Mumbai, India), and “Solution B” contained 2% lignocaine with adrenaline 1:200,000. For the preparation of Solution A, “0.2 mL” of MgSO_4_ was mixed in “1.8 mL” of lignocaine and “1:200,000” adrenaline to make 2 mL of solution. The dental nurse prepared Solution A fresh after receiving the coin from the respective patient.

### 2.2. LA Administration

A single operator (A.S.) administered IANB. Each patient received a maximum of “2 mL” of the solution (1.5 mL for the IANB, 0.25 mL for the long buccal nerve block, and “0.25 mL” for the lingual nerve block). The quadrant of the mouth receiving “Solution A” was assigned to the test group, while the other quadrant receiving “Solution B” was assigned to the control group.

The position of MTM to be removed was evaluated on a preoperative orthopantomogram x-ray and graded as per Pederson's difficulty score. Two independent observers (K.S. and S.G.) quantified the difficulty index. Patients with difficulty index rated as less than or equal to 6 by both the observers were included in the study. All the surgical procedures were carried out following the standard surgical protocol.

### 2.3. Surgical Procedure

A single operator (A.S.) performed all the surgical extractions as per the standard protocol under local anaesthetic coverage under aseptic precautions. The onset and effectiveness of anaesthesia were assessed using objective signs and symptoms of the IANB. Sutures were placed after the extraction once hemostasis was achieved. Following the procedure, patients were prescribed a combination of antibiotics—500 mg amoxicillin with 125 mg potassium clavulanate (Macleods Pharmaceuticals Pvt. Ltd., Mumbai, India)—to be taken three times daily for 3 days. Additionally, they were advised to take an analgesic with anti-inflammatory properties—diclofenac (50 mg) with paracetamol (325 mg) (Win-Medicare Pvt. Ltd., New Delhi, India)—as needed, with a maximum of three tablets per day.

### 2.4. Outcome Assessment

The primary outcomes assessed were the duration of anaesthesia, the time of onset, need for additional injection, and burning sensation as reported by the patient during injection. The secondary outcomes evaluated were postoperative pain and the amount of rescue analgesics consumed. Pain levels were measured using the VAS and mentioned in a proforma provided to the patient before taking any rescue analgesics. Postoperative pain was measured using a 10 cm VAS, with “0” for no pain and “10” for the most severe pain. Patients marked their VAS pain scores on the 1st day, 3rd day, and 7th day postoperatively and recorded the number and time of analgesics intake on proforma. A standardized instruction was given to the patients regarding the reporting of postoperative pain, and a telephonic reminder was sent out to the patients on the evening of the respective days for them to mark the proforma. The proforma was then collected on the day of the follow-up visit for suture removal.

### 2.5. Data Analysis

Data were analysed using Version 20 SPSS (IBM-Corp., 2011, “IBM SPSS” Statistics for Windows, “Version 20.0,” Armonk, NY, United States). A *p* value of less than 0.05 was regarded as statistically significant. The comparison of continuous outcomes was performed using Wilcoxon's signed-rank test owing to the split-mouth design, while the comparison of nominal data (need for additional injection) was done by McNemar's test.

## 3. Results

**Figure 1:**
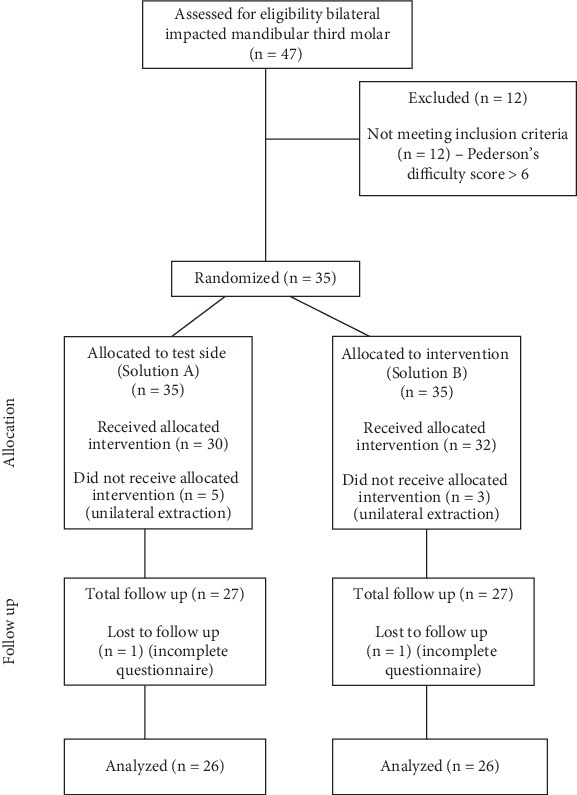
CONSORT flowchart showing the progress of participants included in the randomized controlled trial.

A total of 35 patients were recruited, out of which eight were excluded as only single-side extraction was completed and one patient was excluded due to a discrepancy in the questionnaire. The final analysis included 26 patients (12 males and 14 females) (Figure 1). The average age of the patients was 23.30 ± 3.78 years, ranging from 18 to 30 years. No intraoperative or postoperative complications were reported. The angulation and position of MTM in the test and control sides are mentioned in Table 1.

### 3.1. Intraoperative Outcomes

No significant difference was seen in the time of onset of anaesthesia in test (197.12 ± 26.69) and control sides (196.54 ± 24.93). However, the analgesia duration was significantly longer (*p* < 0.001) in the test side (226.15 ± 10.61) than the control side (179.62 ± 18.7). No significant difference was seen concerning the burning sensation between test and control sides (*p* = 0.059) (Table 2).

The need for additional injections beyond the initially administered IANB was also assessed. Even though 34.6% of patients in the control group needed an additional injection, as compared to 19.2% in the test group, the McNemar test between the two groups failed to show a statistically significant difference (*p* value = 0.289) (Table 3).

### 3.2. Postoperative Outcomes

No significant differences were seen with respect to self-reported mean postoperative pain scores on Days 1, 3, and 7 between MgSO_4_ and control sides (*p* = 0.462, 0.929, and 0.377), respectively. Also, there was no significant difference observed in the mean number of rescue analgesics between test and control sides (*p* = 0.093) (Table 4).

## 4. Discussion

We conducted this split-mouth trial to compare the effect of lignocaine 2% concentration with 1:200,000 adrenaline with or without MgSO_4_ for the administration of IANB before the surgical extraction of impacted MTM. All the participants included in the study had bilateral comparable impacted MTM with a Pederson's difficulty score of ≤ 6.

Pain is the most common adverse event following MTM surgery, mainly due to inflammation caused by tissue injury. This pain arises from pressure on nerve endings due to exudation, which occurs after the release of different inflammatory mediators such as serotonin, bradykinin, and arachidonic acid metabolites. These chemical mediators enhance the sensitivity of pain receptors present at the local site, triggering a pain response [24]. The pain impulse is carried through dorsal horn neurons in the spinal cord to higher brain centers, where it is processed and interpreted [25]. As inflammation progresses, interstitial fluid accumulates because of transudation from damaged vessels and lymphatic drainage obstruction, which is caused by fibrin and fibrinogen clots from plasma and nearby injured vessels, leading to postoperative oedema. Postoperative pain also contributes to trismus observed after MTM surgery. The negative impact of MTM surgery on quality of life (QoL) is reported to be three times higher in patients experiencing oedema, pain, and reduced mouth opening, whether individually or combined, in comparison to asymptomatic patients [26]. Hence, several research works have focused on managing the postsurgical sequelae of MTM surgeries.

Although MgSO_4_ is not used primarily as an analgesic, it positively impacts the efficacy of established analgesics when used as a supplement. The mechanisms of interaction of magnesium in MgSO_4_ and local anaesthetics were first described by Feinstein in 1964. Local anaesthetics inhibit the phospholipid-facilitated transport of calcium across cell membranes. Magnesium, on the other hand, reversibly binds to phospholipid molecules, thereby inhibiting this calcium transport [27]. This is the first mechanism of action of magnesium on enhancing the potency of local anaesthetic. Chronic pain resulting from prolonged nociceptive input can lead to an upregulation of N-methyl-D-aspartate (NMDA) receptors on second-order neurons. Central sensitization enhances the capability of these neurons to respond to painful stimuli (hyperalgesia) and reduces the threshold for pain initiation (allodynia). MgSO_4_ interacts with NMDA receptors, preventing central sensitization induced by peripheral nociceptor stimulation and eliminating established hypersensitivity [28].

MgSO_4_ has reportedly been used for supplementing regional blocks and spinal anaesthesia during various surgical procedures. Intraoperative administration of magnesium during upper limb surgery can lower the consumption of opioids and also, to some extent, VAS pain scores within the first 24 h postsurgery [29]. A study by Sun et al. stated that the addition of 200 mg of MgSO_4_ to ropivacaine 0.25% for a sciatic nerve block provided an analgesic effect comparable to ropivacaine 0.375%. Their findings suggest that even though supplemental MgSO_4_ does not positively impact the analgesic quality, it lowers the amount of local anaesthetic required in sciatic nerve blocks for diabetic toe amputations [30]. Obstetricians frequently administer MgSO_4_ infusions in patients with pre-eclampsia during labor to prevent it from progressing to eclampsia. Additionally, MgSO_4_ has been used as a supplemental analgesic in the postsurgical period after C-section delivery [31]. Narang et al. and Haghighi et al. suggested that adding MgSO_4_ as a supplement to lignocaine increases the duration of the block and accelerates the initiation of motor and sensory blocks during upper limb surgery. However, it also increases the occurrence of transient pain during injection [32, 33]. With regard to the mode of administration, magnesium applied topically to the surgical site has been found to be better than systemic administration in providing postoperative analgesia without inducing any adverse effects [34].

In dental practice, the use of MgSO_4_ has been extensively studied in cases with symptoms of irreversible pulpitis [21, 22, 35]. Trials on patients with irreversible pulpitis in mandibular molars have been associated with a 45%–80% failure rate in obtaining effective anaesthesia using a conventional IA technique [36]. Mekhimar et al. reported that the combination of mepivacaine and MgSO_4_ resulted in a higher success rate for IANB compared to mepivacaine alone; however, there was no statistically significant difference between the groups [35]. Studies done by Shetty et al. and Mousavi et al. focused primarily on assessing the success of IANB with or without the addition of MgSO_4_ to lignocaine [21, 22]. In both their studies, they demonstrated a significant improvement in the success of IANB and pulpal anaesthesia when supplementing lignocaine with MgSO_4_.

There have been limited studies involving the use of MgSO_4_ during the transalveolar extraction of MTM. Jerkovic et al. studied the efficacy of magnesium citrate administered by oral route on postsurgical analgesia and trismus following disimpaction surgery. Their study showed that oral administration of magnesium tablets before and after the surgery significantly decreases both the severity of pain and the degree of trismus in the postoperative phase [37]. Naruenartwongsakul et al. evaluated the anaesthetic efficacy of combining articaine and MgSO_4_ versus articaine alone for IANB in MTM surgery. The addition of MgSO_4_ to articaine significantly enhanced the efficacy and potency of IANB compared to articaine alone in asymptomatic MTM surgery [38].

In our study, we evaluated the need for additional injections during the surgical procedure, which does not necessarily indicate a failure of the block technique. Even though our findings also showed that the group receiving MgSO_4_ had a lesser need for additional injections, it was not found to be statistically significant. The probable explanation for the lesser requirement of additional injections was the significantly longer duration of anaesthesia seen in the MgSO_4_ group. A longer duration of anaesthesia might be a disadvantage, particularly for paediatric or differently abled patients who might inadvertently injure their lips or tongues due to the long duration of numbness. However, long-lasting anaesthesia is effective for analgesia, as it blocks the nociceptive impulse from reaching the CNS [39]. Bupivacaine is a routinely used long-acting duration local anaesthetic, but its safety profile is debated due to potential cardiotoxicity [40]. However, beyond this, no significant differences were observed between the test and control groups in terms of postsurgical analgesia.

Pain is a subjective experience, and its perceived intensity varies among patients. To mitigate this variability, we conducted a split-mouth study with patients having bilateral easy-to-moderate MTM impactions. Each patient was allocated to both the test and control groups, allowing for the assessment of various parameters related to the success and quality of anaesthesia. This approach was aimed at minimizing or eliminating the influence of subjective pain perception and psychological differences between individuals on the results. In this study, the split-mouth design was a strategic choice to control for these confounders, as each patient served as their own control. By including only patients with a similar difficulty index for their impacted mandibular molars and excluding those with pre-existing infections, we aimed to standardize the surgical conditions and minimize the impact of extraneous variables on pain perception and the duration of anaesthesia.

Supplementing local anaesthetics with small doses of MgSO_4_ for blocks can extend the duration of anaesthesia, potentially reducing the side effects associated with high doses of local anaesthetics needed for disimpaction surgery. This deeper level of anaesthesia can help alleviate intraoperative discomfort for the patient. Administration of MgSO_4_ may have sensations of flushing, warmth, nausea, vomiting, and severe toxic side effects such as CNS or respiratory depression; however, these are rare [41]. However, none of these complications was reported in our trial.

To our knowledge, this was the only study conducted by a split-mouth randomized design to assess the potential of MgSO4 for IANB for the removal of symptomatic MTM. The addition of MgSO_4_ to lignocaine with adrenaline extended the duration of anaesthesia and decreased the need for additional injections during the procedure, even though it was not statistically significant. However, the study was limited by excluding patients with a history of allergy to local anaesthetics and was restricted to patients with a conservative range of difficulty in bilateral impacted MTM. Additional research involving symptomatic MTMs or broader inclusion criteria could further clarify the clinical benefits.

## 5. Conclusion

MgSO_4_ with lignocaine for IANB effectively prolongs the duration of anaesthesia. Even though it resulted in a lesser number of additional injections in the MgSO_4_ group, it was found to be statistically not significant. Further, with regard to the number of rescue analgesics and postoperative pain scores, there were no significant differences observed. Lignocaine with MgSO_4_ has limited benefits in extending postoperative analgesia after MTM removal. Additional studies are required to assess its effectiveness in a range of minor oral surgical procedures.

## Figures and Tables

**Table 1:** Distribution of study participants as per the angulation and position of MTM.

	**Test (Solution A)**		**Control (Solution B)**	
	**N**	**%**	**N**	**%**
Angulation				
Mesioangular	7	26.9%	7	26.9%
Horizontal	10	38.5%	11	42.3%
Vertical	9	34.6%	8	30.8%
Position				
A	13	50.0%	15	57.7%
B	13	50.0%	11	42.3%

**Table 2:** Wilcoxon's signed-rank test assessing various intraoperative parameters.

	**Test**		**Control**		**p** ** value**
	**Mean**	**SD**	**Mean**	**SD**	
Time of onset (s)	197.12	26.69	196.54	24.93	0.696; NS
Duration of analgesia (min)	226.15	10.61	179.62	18.70	< 0.001; Sig.
Burning sensation	1.73	0.45	1.92	0.27	0.059; NS

Abbreviations: NS, nonsignificant; Sig., significant.

**Table 3:** McNemar's test to assess the need for additional injection (*p* value of 0.289).

**Need for additional injection**	**Test (Solution A)**	**Control (Solution B)**
Yes	5 (19.2%)	9 (34.6%)
No	21 (80.8%)	17 (65.4%)
Total	26	26

**Table 4:** Wilcoxon's signed-rank test for the assessment of postoperative pain.

	**Test**		**Control**		**p** ** value**
	**Mean**	**SD**	**Mean**	**SD**	
VAS pain—D1	5.35	1.20	5.19	0.85	0.462; NS
VAS pain—D3	2.46	1.21	2.50	0.91	0.929; NS
VAS pain—D7	0.42	0.64	0.58	0.64	0.377; NS
No. of analgesics	6.04	1.15	6.42	1.21	0.093; NS

Abbreviation: NS, nonsignificant.

## Data Availability

The data used to support the findings of this study are available from the corresponding author upon request.

## References

[1] Kim C., Hwang K. G., Park C. J. (2018). Local Anesthesia for Mandibular Third Molar Extraction. *Journal of Dental Anesthesia and Pain Medicine*.

[2] Berge T. I., Bøe O. E. (1994). Predictor Evaluation of Postoperative Morbidity After Surgical Removal of Mandibular Third Molars. *Acta Odontologica Scandinavica*.

[3] Yang F., Gao Y., Zhang L. (2020). Local Anaesthesia for Surgical Extraction of Mandibular Third Molars: A Systematic Review and Network Meta-Analysis. *Clinical Oral Investigations*.

[4] Kambalimath D. H., Dolas R. S., Kambalimath H. V., Agrawal S. M. (2013). Efficacy of 4% Articaine and 2% Lidocaine: A Clinical Study. *Journal of Maxillofacial and Oral Surgery*.

[5] Gregorio L. V., Giglio F. P., Sakai V. T. (2008). A Comparison of the Clinical Anesthetic Efficacy of 4% Articaine and 0.5% Bupivacaine (Both With 1:200,000 Epinephrine) for Lower Third Molar Removal. *Oral Surgery, Oral Medicine, Oral Pathology, Oral Radiology, and Endodontology*.

[6] Jain N., John R. (2016). Anesthetic Efficacy of 4% Articaine Versus 2% Lignocaine During the Surgical Removal of the Third Molar: A Comparative Prospective Study. *Anesthesia, Essays and Researches*.

[7] Krohner R. G. (2003). Anesthetic Considerations and Techniques for Oral and Maxillofacial Surgery. *International Anesthesiology Clinics*.

[8] Seymour R. A., Blair G. S., Wyatt F. A. R. (1983). Post-Operative Dental Pain and Analgesic Efficacy. Part I. *British Journal of Oral Surgery*.

[9] Khademi A., Iranmanesh P., Mosayebi N., Heydari M., Bagherieh S. (2023). Effect of Premedication on the Success of Inferior Alveolar Nerve Block in Patients Diagnosed With Irreversible Pulpitis: An Umbrella Review. *Dental Research Journal*.

[10] Gremillion H. A. (2007). Preface. *Dental Clinics of North America*.

[11] Dray A. (1995). Inflammatory Mediators of Pain. *British Journal of Anaesthesia*.

[12] Jawanda N. K., Shukla A., Singh A., Pentapati K. C., Gadicherla S. (2021). Influence of Lidocaine Including Buprenorphine for Postoperative Analgesia After the Extraction of Mandibular Third Molars: A Randomized Controlled, Double-Blind, Split-Mouth Study. *Scientific World Journal*.

[13] Lawand N. B., Willis W. D., Westlund K. N. (1997). Excitatory Amino Acid Receptor Involvement in Peripheral Nociceptive Transmission in Rats. *European Journal of Pharmacology*.

[14] Tauzin-Fin P., Sesay M., Delort-Laval S., Krol-Houdek M. C., Maurette P. (2006). Intravenous Magnesium Sulphate Decreases Postoperative Tramadol Requirement After Radical Prostatectomy. *European Journal of Anaesthesiology*.

[15] Kara H., Şahin N., Ulusan V., Aydoğdu T. (2002). Magnesium Infusion Reduces Perioperative Pain. *European Journal of Anaesthesiology*.

[16] Tramèr M. R., Glynn C. J. (2002). Magnesium Bier’s Block for Treatment of Chronic Limb Pain: A Randomised, Double-Blind, Cross-Over Study. *Pain*.

[17] Turan A., Memis D., Karamanlioglu B., Güler T., Pamukçu Z. (2005). Intravenous Regional Anesthesia Using Lidocaine and Magnesium. *Anesthesia & Analgesia*.

[18] Lee J. W., Kim M. K., Shin Y. S., Koo B. N. (2007). The Analgesic Effect of Single Dose of Intrathecal Magnesium Sulfate. *Korean Journal of Anesthesiology*.

[19] Ryu J. H., Kang M. H., Park K. S., Do S. H. (2008). Effects of Magnesium Sulphate on Intraoperative Anaesthetic Requirements and Postoperative Analgesia in Gynaecology Patients Receiving Total Intravenous Anaesthesia. *British Journal of Anaesthesia*.

[20] Karaaslan K., Yilmaz F., Gulcu N., Sarpkaya A., Colak C., Kocoglu H. (2008). The Effects of Levobupivacaine Versus Levobupivacaine Plus Magnesium Infiltration on Postoperative Analgesia and Laryngospasm in Pediatric Tonsillectomy Patients. *International Journal of Pediatric Otorhinolaryngology*.

[21] Shetty K. P., Satish S. V., Kilaru K. R., Sardar P., Luke A. M. (2015). Comparison of Anesthetic Efficacy Between Lidocaine With and Without Magnesium Sulfate USP 50% for Inferior Alveolar Nerve Blocks in Patients With Symptomatic Irreversible Pulpitis. *Journal of Endodontia*.

[22] Mousavi S. A., Sadaghiani L., Shahnaseri S., Zandian A., Farnell D. J. J., Vianna M. E. (2020). Effect of Magnesium Sulphate Added to Lidocaine on Inferior Alveolar Nerve Block Success in Patients With Symptoms of Irreversible Pulpitis: A Prospective, Randomized Clinical Trial. *International Endodontic Journal*.

[23] Boutron I., Moher D., Altman D. G., Schulz K. F., Ravaud P., for the CONSORT Group (2008). Extending the CONSORT Statement to Randomized Trials of Nonpharmacologic Treatment: Explanation and Elaboration. *Annals of Internal Medicine*.

[24] Chopra D., Rehan H. S., Mehra P., Kakkar A. K. (2009). A Randomized, Double-Blind, Placebo-Controlled Study Comparing the Efficacy and Safety of Paracetamol, Serratiopeptidase, Ibuprofen and Betamethasone Using the Dental Impaction Pain Model. *International Journal of Oral and Maxillofacial Surgery*.

[25] Cigerim L., Kaplan V. (2019). Evaluation of the Analgesic Efficacies of Dexketoprofen Trometamol and Dexketoprofen Trometamol+ Thiocolchicoside Combinations in the Impacted Third Molar Surgery: Randomised Clinical Trial. *Medicina Oral, Patología Oral Y Cirugía Bucal*.

[26] McGrath C., Comfort M. B., Lo E. C. M., Luo Y. (2003). Changes in Life Quality Following Third Molar Surgery–The Immediate Postoperative Period. *British Dental Journal*.

[27] Feinstein M. B. (1964). Reaction of Local Anesthetics With Phospholipids. *Journal of General Physiology*.

[28] Srebro D., Vuckovic S., Milovanovic A., Kosutic J., Savic Vujovic K., Prostran M. (2017). Magnesium in Pain Research: State of the Art. *Current Medicinal Chemistry*.

[29] Do S. H. (2013). Magnesium: A Versatile Drug for Anesthesiologists. *Korean Journal of Anesthesiology*.

[30] Sun J., Feng X., Zhu Q. (2017). Analgesic Effect of Perineural Magnesium Sulphate for Sciatic Nerve Block for Diabetic Toe Amputation: A Randomized Trial. *PLoS One*.

[31] Shah T. H., Rubenstein A. R., Kosik E. S., Heimbach S. W., Madamangalam A. S. (2018). Parturient on Magnesium Infusion and Its Effectiveness as an Adjuvant Analgesic After Cesarean Delivery: A Retrospective Analysis. *Scientific World Journal*.

[32] Narang S., Dali J. S., Agarwal M., Garg R. (2008). Evaluation of the Efficacy of Magnesium Sulphate as an Adjuvant to Lignocaine for Intravenous Regional Anaesthesia for Upper Limb Surgery. *Anaesthesia and Intensive Care*.

[33] Haghighi M., Soleymanha M., Sedighinejad A. (2015). The Effect of Magnesium Sulfate on Motor and Sensory Axillary Plexus Blockade. *Pain Medicine*.

[34] Demiroglu M., Ün C., Ornek D. H. (2016). The Effect of Systemic and Regional Use of Magnesium Sulfate on Postoperative Tramadol Consumption in Lumbar Disc Surgery. *BioMed Research International*.

[35] Mekhimar A. I., Boghdadi R., Morsy D. A. (2023). Effect of Magnesium Sulphate Added to Mepivacaine Hydrochloride on Inferior Alveolar Nerve Block Success in Patients With Symptomatic Irreversible Pulpitis in Mandibular Molars: A Randomized Clinical Trial. *Advanced Dental Journal*.

[36] Shadmehr E., Aminozarbian M. G., Akhavan A., Mahdavian P., Davoudi A. (2017). Anaesthetic Efficacy of Lidocaine/Clonidine for Inferior Alveolar Nerve Block in Patients With Irreversible Pulpitis. *International Endodontic Journal*.

[37] Jerkovic D., Tadin A., Gavic L., Vladislavic N. Z., Grgic N., Macan D. (2020). Effect of Orally Administered Magnesium on Postoperative Pain Level and Trismus After Surgical Removal of the Lower Third Molars: A Randomized, Double-Blind, Placebo-Controlled Trial. *Clinical Oral Investigations*.

[38] Naruenartwongsakul Y., Powcharoen W., Tachasuttirut K. (2022). Anesthetic Success of Adding Magnesium to Local Anesthetic in Third Molar Surgery. *Proceedings of the 19th International Scientific Conference of the Dental Faculty Consortium of Thailand (DFCT 2022)*.

[39] Camps-Font O., Figueiredo R., Sánchez-Torres A. (2020). Which Is the Most Suitable Local Anaesthetic When Inferior Nerve Blocks Are Used for Impacted Mandibular Third Molar Extraction? A Network Meta-Analysis. *International Journal of Oral and Maxillofacial Surgery*.

[40] Kiuchi M. G., Zapata-Sudo G., Trachez M. M., Ririe D., Sudo R. T. (2011). The Influence of Age on Bupivacaine Cardiotoxicity. *Anesthesia and Analgesia*.

[41] Houlihan S., Decarie D., Benes C. (2016). Magnocaine: Physical Compatibility and Chemical Stability of Magnesium Sulphate and Lidocaine Hydrochloride in Prefilled Syringes. *Journal of Obstetrics and Gynaecology Canada*.

